# The Simplicity of the Brachial Plexus: Common Nerve Roots for Synergistic Function

**DOI:** 10.1097/GOX.0000000000002364

**Published:** 2019-08-08

**Authors:** Brian Mailey, Aladdin H. Hassanein

**Affiliations:** From the Department of Surgery, Institute for Plastic Surgery, Southern Illinois University, Springfield, Ill.; and Division of Plastic Surgery, Indiana University School of Medicine, Indianapolis, Ind.

The upper extremity moves in coordinated motions to accomplish complex tasks.^1,2^ This requires contraction of multiple muscles in unison for actions to occur in a natural, seamless fashion.^2^ For example, as one brings one's hand to his or her mouth, the shoulder externally rotates, extends and adducts, while the hand supinates and the elbow flexes. The muscles required to perform this movement are innervated by various terminal nerve branches (eg, suprascapular, axillary, lateral pectoral nerve, musculocutaneous, radial nerve), but fibers all originate from the same C5-6 nerve roots. Less natural motions require more intentional action of the thorax, shoulder, and elbow. The purpose of this article is to examine the organization of the brachial plexus in one simple, unifying theme: muscles involved in synergistic function share a common nerve root, despite their terminal branch.

The brachial plexus originates as 5 ventral roots from C5 to T1 and terminates as 5 motor branches that power the upper extremity. Between the roots and branches, the plexus becomes 3 trunks, 6 divisions, and 3 cords. Along this course, the plexus gives off 12 other terminal branches. The intervening convolution between the roots and branches, at first glance, would seem unnecessary. The crossing divisions and contributions from each cord provide complexity in anatomy that cannot be explained by redundancy, as loss of a single nerve can lead to devastating loss of function.

The anatomical intricacies, however, can be explained as a highway system for nerve roots to arrive at target muscles, with multiple muscles working together to perform 1 coordinated motion. The intervening trunks, divisions, and cords can be thought of as simply a system to deliver axons from a similar nerve root to terminal branches that reach the end target muscle of interest. Each of these motions occurs as a result of various muscles contracting together and are all innervated by similar nerve roots, despite variations in branches.

Cadaveric dissections demonstrate variations in innervation patterns. The triceps receives nerve fibers from the radial nerve, axillary nerve, and/or even branches off the ulnar nerve.^3,4^ Anatomic discrepancies can also been seen for the extensor carpi radialis brevis between the superficial branch of the radial nerve (55%), posterior interosseous nerve (2nd most common), and radial nerve.^5^ Despite the variability in terminal nerve branch, the derivative nerve root remains consistent.

The more proximal and distal muscles are primarily innervated by the higher and lower nerve roots, respectively. However, this is not uniformly true. For example, the pectoralis major is the only muscle with 2 nerves directly off the brachial plexus and innervation from every nerve root (lateral pectoral nerve C5, C6, C7 and medial pectoral nerve C8, T1). The lack of innervation order can also be seen for the finger extensors.

The brachial plexus is a complex network of structure and function whose layout can be simplified into axons going from point A (nerve root) to point B (synergistic muscle) with the intervening trunks, cords and divisions being a system to deliver these axons to synergistic muscles that co-contract in unison. As the philosophical principle, Occam's razor, states, the simplest solution tends to be correct. This may very well also be the case for the brachial plexus.
